# Protective Effect of Hyperprolactinemia on Oxidative Stress in Patients with Psychotic Disorder on Atypical Antipsychotics Risperidone and Paliperidone: A Cross-Sectional Study

**DOI:** 10.3390/biomedicines12071418

**Published:** 2024-06-26

**Authors:** Milena Stojkovic, Mirjana Jovanovic, Vladimir Jakovljevic, Vladimir Zivkovic, Natasa Djordjevic, Aleksandar Kocovic, Marina Nikolic, Aleksandra Stojanovic, Natasa Minic, Vesna Ignjatovic, Vladimir Vukomanovic, Danijela Nastic, Natasa Zdravkovic, Olivera Radmanovic, Milan Djordjic, Sasa Babic, Branimir Radmanovic

**Affiliations:** 1 Department of Psychiatry, Faculty of Medical Sciences, University of Kragujevac, 34000 Kragujevac, Serbia; drminjaj@yahoo.com (M.J.); natasaminic92@gmail.com (N.M.); biokg2005@yahoo.com (B.R.); 2 Psychiatric Clinic, University Clinical Center Kragujevac, 34000 Kragujevac, Serbia; drsasab@gmail.com; 3 Department of Physiology, Faculty of Medical Sciences, University of Kragujevac, 34000 Kragujevac, Serbia; drvladakgbg@gmail.com (V.J.); vladimirziv@gmail.com (V.Z.); marina.rankovic.95@gmail.com (M.N.); 4 Center of Excellence for the Study of Redox Balance in Cardiovascular and Metabolic Disorders, University of Kragujevac, 34000 Kragujevac, Serbia; vranicaleksandra90@gmail.com; 5 Department of Human Pathology, 1st Moscow State Medical, University IM Sechenov, 119991 Moscow, Russia; 6 Department of Pharmacology, 1st Moscow State Medical, University IM Sechenov, 119991 Moscow, Russia; 7 Department of Pharmacology and Toxicology, Faculty of Medical Sciences, University of Kragujevac, 34000 Kragujevac, Serbia; natashadj2002@yahoo.com; 8 Department of Pharmacy, Faculty of Medical Sciences, University of Kragujevac, 34000 Kragujevac, Serbia; 9 Department of Nuclear Medicine, Faculty of Medical Sciences, University of Kragujevac, 34000 Kragujevac, Serbia; vesnaivladaignjatovic@gmail.com (V.I.); vukomanovic@gmail.com (V.V.); 10 Institution for Accommodating Adults “Male Pcelice”, 34000 Kragujevac, Serbia; danijelanastic@yahoo.com; 11 Department of Internal Medicine, Faculty of Medical Sciences, University of Kragujevac, 34000 Kragujevac, Serbia; natasasilvester@gmail.com; 12 Clinic of Gastroenterohepatology, University Clinical Center Kragujevac, 34000 Kragujevac, Serbia; 13 Clinic for Rheumatology and Allergology, University Clinical Center Kragujevac, 34000 Kragujevac, Serbia; olja.radmanovic@gmail.com; 14 Department of Communication Skills, Ethics, and Psychology, Faculty of Medical Sciences, University of Kragujevac, 34000 Kragujevac, Serbia; mcpikac@gmail.com

**Keywords:** hyperprolactinemia, oxidative stress, prolactin, antipsychotics, risperidone, paliperidone

## Abstract

Several studies indicate the impact of antipsychotics like risperidone and paliperidone on oxidative stress parameters, yet data remain inconsistent. We investigated the link between these medications, hyperprolactinemia (HPRL), and oxidative stress. This study was conducted at the Psychiatry Clinic, University Clinical Center, Kragujevac, between November 2022 and August 2023. Inclusion criteria comprised diagnosed psychotic disorders from the ICD-10-based F20-F29 spectrum and clinical stability on risperidone/paliperidone for ≥12 weeks with no recent dose adjustments. Exclusion criteria included pregnancy, breastfeeding, relevant medical conditions, or co-therapy with prolactin-secreting drugs. Data encompassed drug choice, administration method, therapy duration, and daily dose. Prolactin (PRL) levels, oxidative stress parameters (TBARS, H_2_O_2_, O_2_^−^, NO_2_^−^), and antioxidant system (CAT, GSH, SOD) were assessed. Of 155 subjects, women exhibited significantly higher PRL levels (*p* < 0.001) and symptomatic HPRL (*p* < 0.001). Drug choice and regimen significantly influenced TBARS (*p* < 0.001), NO_2_^−^ (*p* < 0.001), O_2_^−^ (*p* = 0.002), CAT (*p* = 0.04), and GSH (*p* < 0.001) levels. NO_2_^−^ levels were affected by drug dose (*p* = 0.038). TBARS (*p* < 0.001), O_2_^−^ (*p* < 0.001), and SOD (*p* = 0.022) inversely correlated with PRL levels, suggesting PRL’s protective role against oxidative stress. The female sex association with higher PRL levels implies additional factors influencing PRL’s antioxidant role. Antipsychotic choice and dosage impact PRL and oxidative stress markers, necessitating further exploration.

## 1. Introduction

Risperidone and its active metabolite paliperidone are atypical antipsychotics used to treat psychiatric disorders such as schizophrenia, bipolar I acute, manic or mixed episodes, and autism spectrum disorders [1,2,3,4]. They act by antagonizing the effect of certain neurotransmitters in the brain, particularly dopamine and serotonin.

PRL is a hormone produced by the pituitary gland in the brain. It plays a crucial role in the female reproductive system, such as promoting milk production after childbirth, but also has other functions in both men and women [5]. Elevated levels of PRL have been observed in a variety of medical conditions, including psychiatric disorders [5], and can be either asymptomatic or associated with several signs and symptoms that can seriously affect the quality of life [2,5,6]. Moreover, it has been previously suggested that drug-naïve people with a first episode have higher blood concentrations of prolactin than healthy subjects, supporting the hypothesis that abnormal prolactin secretion may be involved in the onset of psychoses regardless of antipsychotic treatment [7]. One of the most significant side effects of risperidone and paliperidone is hyperprolactinemia (HPRL), which develops as a result of dopaminergic D2 tuberoinfundibular receptor blockade [2,3,4].

Oxidative stress is a condition characterized by an imbalance between the production of reactive oxygen species (ROS) and the body’s ability to neutralize them through antioxidants. The consequences of oxidative stress are damage to cells, proteins, lipids, and DNA, which can contribute to the development and progression of various diseases, including neurodegenerative and cardiovascular disorders, as well as tumors [8,9]. While there is strong evidence that ROS may play an important role in the pathophysiology of schizophrenia [10], several preclinical and clinical studies have demonstrated that certain typical and atypical antipsychotics, including risperidone and paliperidone, also affect oxidative stress and alter antioxidant enzyme levels [11].

The available literature suggests the possibility of a significant association among atypical antipsychotics, HPRL, and oxidative stress [10,11,12], yet the data are insufficient and inconsistent. Reports have indicated that PRL acts as a safeguard against glutamate-induced apoptosis, increasing the production and activity of SOD, reducing lipid peroxidation in hippocampal neurons exposed to glutamate excitotoxicity, and having anti-inflammatory effects. On the other hand, some results suggest that the effects of PRL seem to depend on the serum concentration: low concentrations of PRL inhibit cytotoxicity and oxidative stress, while high PRL concentrations induce the opposite effects [7].

Therefore, our study aimed to examine if there is any connection between risperidone/paliperidone, increased PRL levels, and ROS parameters in patients diagnosed with psychotic disorders. Our second objective was to evaluate and compare the impact of the applied dose, method of administration, and duration of treatment on the levels of PRL and oxidative stress markers.

## 2. Materials and Methods

### 2.1. Study Subjects

This study was conducted at the Psychiatry Clinic, University Clinical Center, Kragujevac, Serbia, and included patients diagnosed with psychotic disorder from the ICD-10-based F20–F29 spectrum. All study subjects had to be clinically stable (on risperidone or paliperidone treatment for at least 12 weeks, with no dose adjustment during the last 4 weeks); older than 18 years; able to understand the nature of the study; sign informed consent; live in a stable social environment; capable of providing reliable data. On the other hand, pregnancy or breastfeeding; diagnosis of other mental conditions (including alcoholism and substance abuse); congenital intellectual deficit, organic brain disorders, hypothyroidism, diabetes mellitus, or liver disease; or cotherapy with prolactin-secreting drugs, other antipsychotics, or drugs that affect sexual activity were considered the exclusion criteria. This research was approved by the Ethics Committee of the University Clinical Center, Kragujevac (Decision No 01/22-339) and was conducted from November 2022 to August 2023.

### 2.2. Data and Sample Collection

For each patient, the following treatment-related data were obtained (based on the interview with the patient as well as from the hospital medical records): (1) the choice of the drug and the method of its administration (oral risperidone (OR); two-week depot risperidone, i.e., long-acting injections of risperidone (LAI-R); monthly depot paliperidone, i.e., long-acting injections of paliperidone monthly (LAI-Pm); three-month depot paliperidone, i.e., long-acting injections of paliperidone quarterly (LAI-Pq)); (2) the duration of therapy (less than 6 months; from 6 months to a year; more than a year); and (3) the dose of the drug per day (2–4 mg of risperidone (low), 5–6 mg of risperidone (medium), and 7–8 mg of risperidone (high)).

To assess hormonal and biochemical status, 20 mL fasting blood samples were obtained from the study participants; test tubes with citrate buffer (3.2% sodium citrate; 109 mmol/L) were used for sampling. The blood was centrifuged at 3000 rpm for 10 minutes, and the plasma was separated. Erythrocyte washing was carried out as follows: cold saline was added in a ratio of 1:3 to the test tube and centrifuged again under the same conditions 3 times. After the third time, the supernatant was first removed, then 1 mL of erythrocytes was separated, and 3 mL of cold distilled water was added. Plasma and erythrocyte samples were stored at −20 °C until analyses.

### 2.3. Prolactin Level Measurement

Blood samples were obtained to evaluate the serum level of prolactin (PRL). Prolactin was assayed with immunoradiometric assay (IRMA) systems. We used a Prolactin IRMA kit (Beckman Coulter Company—Prague, Czech Republic), with reference ranges of 3.06–26.9 ng/mL, 2.64–37.2 ng/mL, and 2.77–14.4 ng/mL in men, women before menopause, and women after menopause, respectively [13]. The concentration of prolactin was measured with a gamma counter (WALLAC WIZARD 1470 Automatic, Perkin Elmer Life Sciences, Wallac Oy, Turku, Finland). Based on the PRL level and the presence of clinical signs and symptoms, study participants were classified as having (1) symptomatic HPRL, or (2) asymptomatic HPRL, or (3) displaying PRL level within the reference range.

### 2.4. Oxidative Stress Measurements

The parameters of oxidative stress and antioxidant protection were measured spectrophotometrically in plasma and erythrocytes (Specord S-600 Analytik, Jena, Germany) at the Laboratory for Experimental Cardiovascular Physiology, Faculty of Medical Sciences, University of Kragujevac.

The degree of lipid peroxidation, i.e., ROS, in plasma was estimated by the method previously reported by Ohkawa et al. [14]. Briefly, we used TBARS as an index of lipid peroxidation: 0.4 mL 1% thiobarbituric acid (TBA) in 0.05 NaOH was mixed with 0.8 mL of plasma, incubated at 100 °C for 15 min, and measured at 530 nm, with distilled water used as a blank. The determination of H_2_O_2_ in plasma was based on the oxidation of phenol red (PR) in the presence of horseradish peroxidase (HRPO), as described by Pick et al. [15]. Then, 200 μL of plasma sample with 800 μL of PR solution and 10 μL of HRPO were combined 1:20. The level of H_2_O_2_ was then determined based on the absorbance measured at 610 nm. NO_2_^−^ levels in plasma were determined as an index of nitric oxide production with Griess reagent, as explained by Green et al. [16]. In short, a total of 0.1 mL of 3N PCA (perchloric acid), 0.4 mL of 20 mM EDTA (ethylenediaminetetraacetic acid), and 0.2 mL of plasma were put on ice for 15 min, then centrifuged for 15 min at 6000 rpm. After pouring off the supernatant, 220 μL of K_2_CO_3_ was added, nitrites were measured at 550 nm, while distilled water was used as a blank probe. The levels of O_2_^−^ in plasma samples were measured using NBT reaction in TRIS buffer combined with plasma samples and read at 530 nm [17].

To determine the enzymes of AOS, isolated erythrocytes were washed three times with three vol-mixtures of 0.9% NaCl. Hemolyzate prepared according to McCord and Fridovich [18], containing about 50 g of Hb/L, was used to measure CAT activity. To determine the activity of CAT, lysates were diluted in distilled water (1:7 *v*/*v*) and treated with chloroform ethanol (0.6:1 *v*/*v*) to remove hemoglobin [19]. Then, 50 μL CAT buffer, 100 μL sample, and 1 mL 10 mM H_2_O_2_ were added to the samples. Detection was performed at 360 nm, and distilled water was used as a blank probe [20]. SOD activity was determined with the epinephrine method. A total of 100 μL of epinephrine was added to a mixture of 100 μL of lysates and 1 mL of bicarbonate buffer. The measurement was made at 470 nm. The level of GSH was determined based on glutathione oxidation with 5,5 dithio-bis-6,2-nitrobenzoic acid using the Beutler method; the concentration is expressed in nanomoles per milliliter of erythrocyte [21].

### 2.5. Statistical Analysis

SPSS version 26 (IBM, Armonk, NY, USA) was used for statistical analysis. The Kolmogorov–Smirnov test was used to test the normality of the data, and the values are expressed as mean ± standard error of the mean. To test the differences between groups, one-way ANOVA or Student’s *t*-test was used. The statistical significance threshold was set at 0.05.

## 3. Results

A total of 155 subjects were included in the study, of which 101 (65.2%) were men, and 54 (34.2%) were women. General data about the test subjects are presented in Table 1.

The female sex was associated with significantly higher PRL levels (female 42.21 ± 23.28 ng/mL vs. male 19.33 ± 11.01 ng/mL; t(153): −8.413, *p* < 0.001), and the difference remained significant after stratification according to PRL groups (t(153): −6.335, *p* < 0.001). Among the patients with HPRL, the symptomatic form was statistically more frequent in women as compared to men (69.7% vs. 30.3%, χ^2^ = 32.307, *p* < 0.001). The choice of the treatment regimen (χ^2^ = 5.222, *p* = 0.156) or the duration of therapy (χ^2^ = 0.643, *p* = 0.725) did not differ based on sex.

There was a statistically significant difference in the level of PRL depending on the choice and the method of drug administration (F = 4.601, *p* = 0.004), with the highest and lowest values identified in the OR and LAI-Pq groups, respectively (41.16 ± 12.73 ng/mL vs. 41.38 ± 11.436 ng/mL, Tukey’s HSD = 14.15, *p* = 0.003). Neither the duration of the treatment (F = 0.410, *p* = 0.664) nor the dose of the drug (F = 0.534, *p* = 0.587) affected the level of PRL.

There was no association observed between age and ROS and AOS parameters.

**Figure 1:**
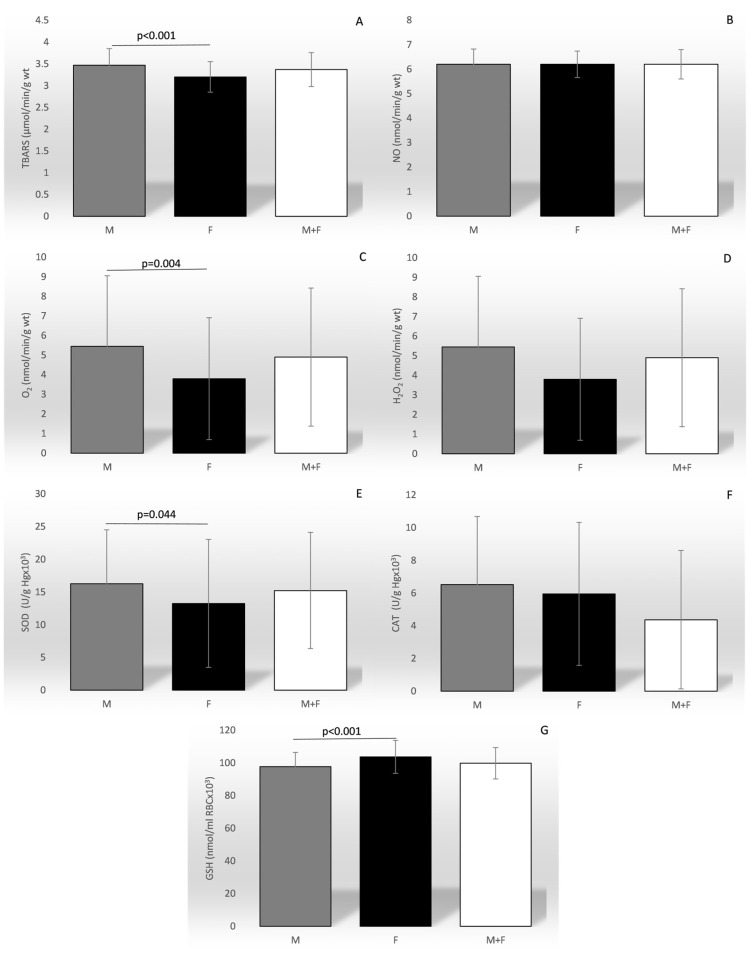
Comparison of the mean values of the parameters of oxidation stress between sexes and in total. TBARS—index of lipid peroxidation (graph (**A**)); NO—nitric oxide (graph (**B**)); O_2_^−^—superoxide anion radical (graph (**C**)); H_2_O_2_—hydrogen peroxide (graph (**D**)); SOD—superoxide anion radical (graph (**E**)); CAT—catalase (graph (**F**)); GSH—reduced glutathione (graph (**G**)).

A sex-based comparison of ROS and AOS revealed significantly higher mean values of TBARS, O_2_^−^, and SOD, but lower GSH, in men (Figure 1).

**Figure 2:**
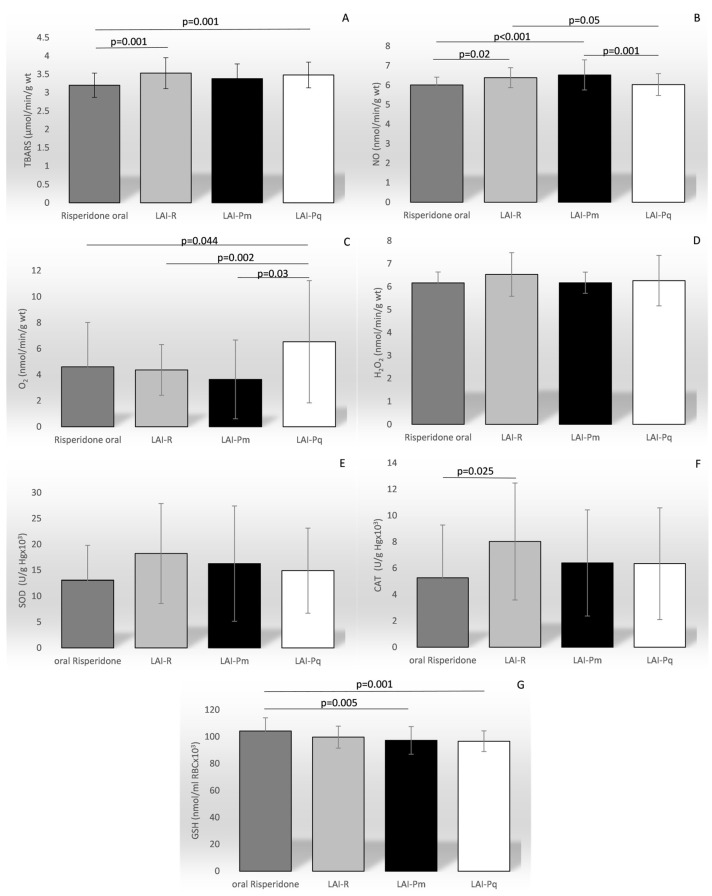
Comparison of the mean values of the parameters of oxidation stress between different therapy regimens. TBARS—index of lipid peroxidation (graph (**A**); NO—nitric oxide (graph (**B**)); O_2_^−^—superoxide anion radical (graph (**C**)); H_2_O_2_—hydrogen peroxide (graph (**D**)); SOD—superoxide anion radical (graph (**E**)); CAT—catalase (graph (**F**)); GSH—reduced glutathione (graph (**G**)).

The drug choice and the treatment regimen significantly affected the level of most of the ROS and AOS parameters (Figure 2), including TBARS (F = 7.171, *p* < 0.001), NO level (F = 7.876, *p* < 0.001), O_2_^−^ (F = 5.166, *p* = 0.002), CAT (F = 2.717, *p* = 0.04), and GSH (F = 6.478, *p* < 0.001). There was no association observed between the duration of the therapy and any of the ROS or AOS parameters.

**Figure 3:**
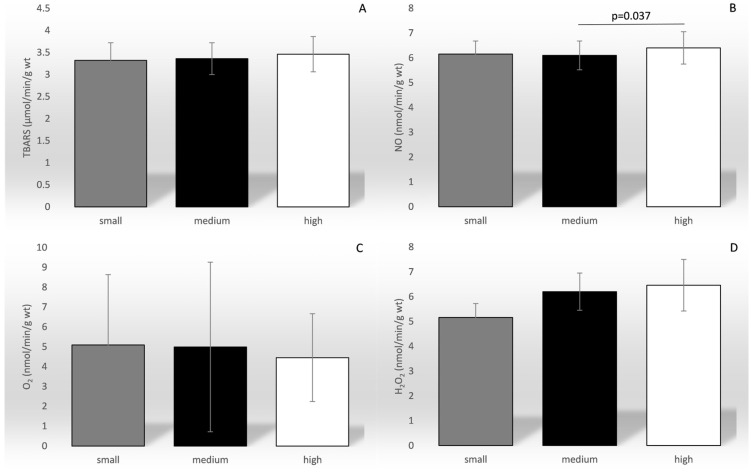
Comparison of the mean values of the parameters of oxidation stress between different categories of drug dosage. TBARS—index of lipid peroxidation (graph (**A**)); NO—nitric oxide (graph (**B**)); O_2_^−^—superoxide anion radical (graph (**C**)); H_2_O_2_—hydrogen peroxide (graph (**D**)). The dose of the drug was categorized as small (2–4 mg/day), medium (5–6 mg/day), and high (7–8 mg/day).

Of all the ROS parameters, only the level of NO was significantly affected by the drug dose (F = 3.330, *p* = 0.038; Figure 3).

The levels of TBARS (r = −0.630, *p* < 0.001), O_2_^−^ (r = −0.368, *p* < 0.001), and SOD (r = −0.184, *p* = 0.022) correlated negatively with PRL levels, while no significant association was observed for the other ROS and AOS parameters. 

**Figure 4:**
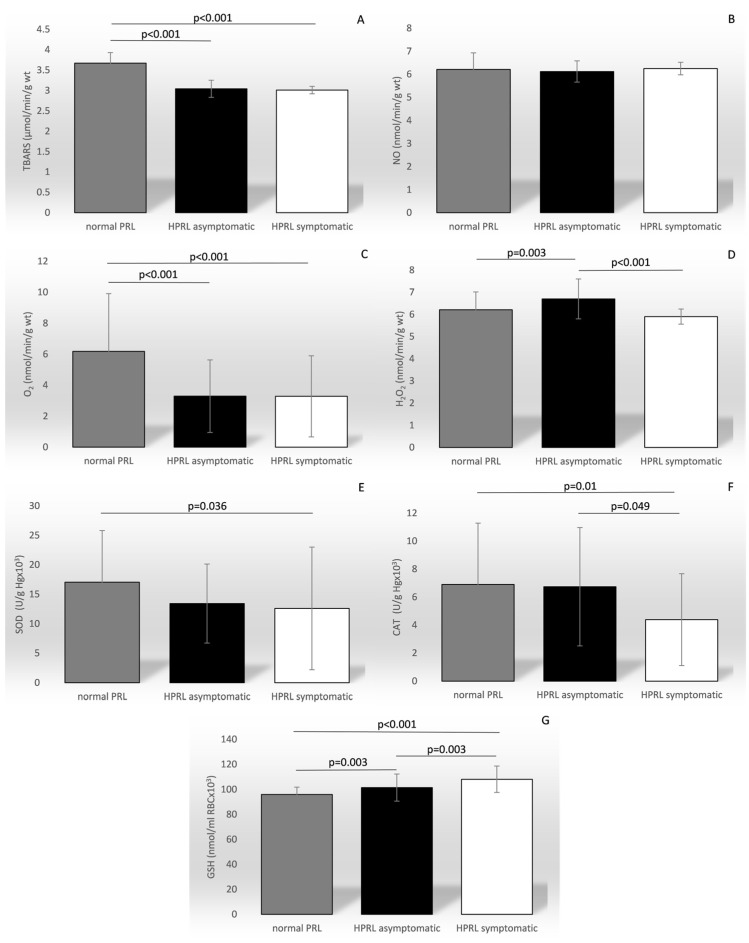
Comparison of the mean values of the parameters of oxidation stress between groups categorized by the level of prolactin and symptom manifestation. TBARS—index of lipid peroxidation (graph (**A**)); NO—nitric oxide (graph (**B**)); O_2_^−^—superoxide anion radical (graph (**C**)); H_2_O_2_—hydrogen peroxide (graph (**D**)); SOD—superoxide anion radical (graph (**E**)); CAT—catalase (graph (**F**)); GSH—reduced glutathione (graph (**G**)); PRL—prolactin; HPRL—hyperprolctinemia.

When the patients were stratified according to PRL level, the mean levels of all of the ROS and AOS parameters, except for NO, differed among the PRL groups, as presented in Figure 4: TBARS (F = 159.445, *p* < 0.001), O_2_^−^ (F = 15.421, *p* < 0.001), H_2_O_2_ (F = 10.187, *p* < 0.001), SOD (F = 4.178, *p* = 0.017), CAT (F = 4.607, *p* = 0.011), and GSH (F = 25.817, *p* < 0.001).

There was a statistically significant difference in the prolactin level depending on whether the drug was administered orally or intramuscularly (T = 3.668; *p* < 0.001). The average value of prolactin in the group treated with the oral preparation was 35.26 ± 19.59, while in the group treated with the intramuscular preparation, it was 23.44 ± 18.61

**Figure 5:**
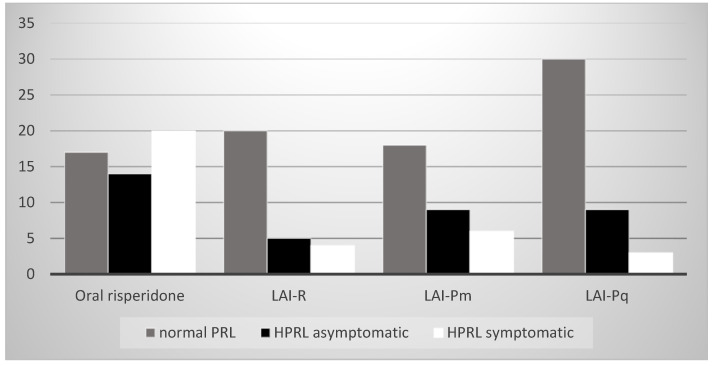
The association of the drug used in therapy, level of prolactin, and symptom manifestation. PRL—prolactin; HPRL—hyperprolctinemia.

There was a statistically significant difference in the value of the prolactin level depending on the drug used in the therapy (χ^2^ = 21.098; *p* = 0.002), i.e., oral risperidone most often led to symptomatic and asymptomatic hyperprolactinemia. The association of the drug used in therapy, the level of prolactin, and symptom manifestation is presented in Figure 5.

## 4. Discussion

In the present study, we analyzed PRL levels, reactive oxygen species, and enzymes of the antioxidant system in patients with psychosis treated with risperidone and paliperidone. Our results indicate a protective role of increased PRL levels against markers of oxidative stress. In addition, our study shows that both sex and the drug therapy regimen affect the levels of PRL, ROS, and AOS parameters.

According to several studies, pituitary alterations are likely involved in schizophrenia and psychosis onset [7], and patients with schizophrenia possibly have an exaggerated prolactin response to stress, as a consequence of the genetic variations in prolactin or autoimmune mechanisms [22,23]. Riecher-Rössler et al. (2013) (REF) suggested that stress increases PRL secretion and triggers dopamine release via a feedback mechanism. The increase in dopamine transmission mediates the link between stress and psychosis in the tuberoinfundibular pathway. Hyperprolactinemia was found in one-third of patients in antipsychotic-naïve at-risk mental states as well as in one-third of patients with first-episode psychosis [24].

Cavaleri et al. (2024) had similar results for drug-naïve people with first psychotic episodes regarding their altered ACTH, PRL, and TSH blood concentrations, supporting the hypothesis of abnormal anterior pituitary hormone secretion [7]. Moreover, stress has a stronger effect on women than on men in emerging psychosis [25]. However, it remains unclear why some patients have normal levels of PRL, and increased PRL produces similar changes in the mesolimbic pathway such as in tuberoinfundibular.

The influence of PRL on oxidative stress has been previously examined in several preclinical and clinical studies. Most of the earlier reports suggested the protective role of PRL, consistent with our findings that subjects with PRL levels within the reference range, as compared to patients with either symptomatic or asymptomatic HPRL, display significantly higher levels of ROS. Rivero-Segura et al. [26] demonstrated that PRL protects against glutamate-induced apoptosis by preserving mitochondrial function and increasing the production and activity of SOD, thus decreasing lipid peroxidation in the hippocampal neurons exposed to glutamate excitotoxicity, with positive effects on anti-inflammatory cytokines IL-10 and IL-4. García et al. [27] also showed that PRL reduced ROS production and protected against oxidative damage by promoting cell survival and reducing apoptosis, with a protective effect mediated by an increase in the production of the antioxidant GSH. Most recently, Ramos-Martinez et al. [28] observed a positive correlation of PRL with antioxidant enzymes. On the other hand, the dual role of PRL, which seems to be dependent on the serum concentration, has been described as well: low concentrations of PRL inhibit cytotoxicity and oxidative stress, while high PRL concentrations induce the opposite effects [29].

As previously mentioned, ROS have a very short life span and are usually neutralized by antioxidants before damage occurs [8]. SOD dismutates O_2_^·−^ to H_2_O_2_ and O_2_, followed by H_2_O_2_ being promptly removed by CAT or GPx. Therefore, high SOD activity results in increased H_2_O_2_ production, which must be accompanied by increased GPx and/or CAT activity to prevent damage. GPx eliminates H_2_O_2,_ lipid peroxides, and hydroxyl radicals into nontoxic forms via reduction that utilizes GSH. This is followed by the concomitant oxidation of reduced GSH into the oxidized form, glutathione disulfide (GSSG), and glutathione reductase recycles GSSG to GSH [29]. Once oxidized, GSSG can be reduced back by glutathione reductase using NADPH as an electron donor, or GSH can be generated by de novo synthesis [30,31,32]. Based on the available literature, it seems that PRL may interfere with both regulatory pathways at different levels [28]. In our study, subjects with PRL within the reference range demonstrated the highest level of TBARS, O_2_^−^ and SOD as compared to patients with HPRL, while H_2_O_2_ and CAT levels were highest in the patients who were asymptomatic, and GSH was highest in the symptomatic HPRL group. High TBARS, as a byproduct of lipid peroxidation [33], together with high levels of O_2_^−^, links higher oxidative stress to lower PRL levels, while increased levels of H_2_O_2_ suggest the higher antioxidant activity of SOD in the presence of higher PRL concentrations. Similarly, higher levels of SOD in subjects with reference PRL are indicative of a higher need for antioxidant activity, while increased CAT and GSH in HPRL could be explained by higher H_2_O_2_ production.

The association of psychiatric disorders with oxidative stress is already well known. Studies have shown that individuals with psychiatric disorders, including schizophrenia, bipolar disorder, and depression, may have higher oxidative stress marker levels than healthy individuals [34]. The exact mechanisms linking oxidative stress and psychiatric disorders are not fully understood, and chronic inflammation, altered antioxidant systems, and mitochondrial dysfunction are among the factors that have been offered as explanations [34,35]. Similarly, it is yet unknown whether oxidative stress is the primary cause of the disease, or whether it occurs secondarily under the influence of environmental factors or long-term treatment. On the one hand, it has been shown that subjects with schizophrenia experience endogenous oxidative stress, which can trigger the initiation of symptoms [8]. On the other hand, antipsychotic treatment may trigger oxidative imbalance; however, both typical and atypical antipsychotics vary significantly in their effects on redox balance [36].

Several preclinical studies indicate that the typical antipsychotics most often cause oxidative stress by reducing the activity of the antioxidant enzymes SOD and CAT and increasing lipid peroxidation [10,37]. However, it was previously suggested that atypical antipsychotics, such as risperidone and paliperidone, may not have any effects on antioxidant enzyme levels during acute treatment, but long-term treatment can contribute to pro-oxidant processes [10,38]. Opposite to the typical antipsychotics, which decrease reduced GSH, atypical antipsychotics lead to an increase in GSH levels, which could be connected with the improvement in symptoms [10]. It has been reported that risperidone upregulates SOD gene expression and downregulates p-75 neurotropin receptor mRNA levels, suggestive of neuroprotective potential in terms of free radical production in patients schizophrenia [10]. In our study, the level of lipid peroxidation, estimated by TBARS, as well as the level of NO were the lowest in patients on oral risperidone treatment and the highest in those on LAI-R and LAI-Pm. On the other hand, both SOD and CAT levels were lower while GSH was significantly higher on risperidone as compared to other treatment options. In addition, we found NO to be significantly higher in patients on the highest daily dose, while a similar trend of a positive correlation of the drug dose with the level of ROS parameters was observed for TBARS and H_2_O_2_ as well. One of the possible explanations might include the impact of PRL on ROS, as its highest levels, apparently protective against oxidative stress, were observed in patients on oral risperidone treatment. It is worth noting that the duration of the treatment did not significantly affect the level of oxidative stress, regardless of longer treatments being associated with older age, otherwise known as the accumulation of oxidative damage [39]. However, the duration of treatment did not affect the PRL level either, which is in line with its proposed role in oxidative stress protection. On the other hand, higher PRL levels in women, consistent with the physiological difference between the sexes, contradict our findings of higher TBARS and O_2_^−^ and lower GSH in women as compared to men, questioning the significance of PRL in oxidative stress attenuation and possibly introducing other factors that might play a role in the processes.

It should be noted that our study has certain limitations, including its cross-sectional design, relatively small sample size, the lack of a group on oral paliperidone (which is currently unavailable in our country), as well as the lack of data on the levels of PRL, ROS, and AOS parameters prior to treatment. Additional prospective studies designed to overcome these biases would be crucial to assess the potential clinical utility of our findings.

## 5. Conclusions

In conclusion, we report the protective role of increased PRL levels against oxidative stress in patients with psychosis treated with risperidone and paliperidone. Yet, the association between female sex and higher PRL levels, in the context of female-related increased ROS and decreased AOS markers, suggests additional factors might alter the proposed antioxidant role of PRL. The choice of antipsychotics and the drug dosage seem to affect both PRL and oxidative stress marker levels, and the nature of their interactions is yet to be elucidated.

## Figures and Tables

**Table 1:** General data about the test subjects.

Variable	Group	Data	Statistics; *p* Value
**Total number**		155	
**Sex (n (%))**	Male	101 (65.2%)	
	Female	54 (34.2%)	
**Age (M ± SD)**		41.7 ± 11.9 years	
**Age by sex** **(M ± SD)**	Male	39.5 ± 11.4	t = −3.190, *p* = 0.002
	Female	45.7 ± 11.9	
**PRL level** **(M ± SD)**		27.41 ± 19.70 ng/mL	
**PRL level by sex** **(M ± SD)**	Male	19.33 ± 11.01	t = −6.914, *p* < 0.001
	Female	42.21 ± 23.28	
**Number of patients by PRL group** **(n (%))**	Normal PRL	85 (54.8%)	
	Asymptomatic HPRL	37 (23.9%)	
	Symptomatic HPRL	33 (21.3%)	
**PRL level by PRL group (M ± SD)**	Normal PRL	14.08 ± 6.00 ng/mL	F = 142.559; *p* < 0.001
	Asymptomatic HPRL	38.18 ± 11.78 ng/mL	
	Symptomatic HPRL	53.01 ± 19.97 ng/mL	
**Age by PRL group (M ± SD)**	Normal PRL	40.3 ± 11.4 years	F = 1.367; *p* = 0.258
	Asymptomatic HPRL	42.6 ± 12.8 years	
	Symptomatic HPRL	44.1 ± 12.1 years	
**Number of patients by drug form** **(n (%))**	Oral risperidone	51 (32.9%)	
	LAI-R	29 (18.7%)	
	LAI-Pm	33 (21.3%)	
	LAI-Pq	42 (27.1%)	
**Age by drug form** **(M ± SD)**	Oral risperidone	41.16 ± 1.8 years	F = 0.099; *p* = 0.961
	LAI-R	42.25 ± 2.8 years	
	LAI-Pm	42.18 ± 2.0 years	
	LAI-Pq	41.38 ± 1.8 years	
**Duration of therapy** **(n (%))**	Under 6 months	42 (27.1%)	
	6–12 months	25 (16.1%)	
	Over 12 months	88 (56.8%)	
**Age by duration of therapy** **(M ± SD)**	Under 6 months	38.26 ± 12.8years	F = 6.210; *p* = 0.003
	6–12 months	37.33 ± 11.4years	
	Over 12 months	44.50 ± 10.9 years	

M—mean; SD—standard deviation; PRL—prolactine; HPRL—hyperprolactinemia; LAI-R—long-acting injections of risperidone; LAI-Pm—long-acting injections of paliperidone monthly; LAI-Pq—long-acting injections of paliperidone quarterly.

## Data Availability

The datasets used and/or analyzed in the present study are available from the corresponding author upon request. The data are not publicly available due to privacy and ethical restrictions.
